# Re: The pitfalls of focusing on cardiovascular disease mortality to explain differences in life expectancy

**DOI:** 10.1007/s10654-024-01102-y

**Published:** 2024-02-12

**Authors:** Domantas Jasilionis, Alyson van Raalte, Sebastian Klüsener, Pavel Grigoriev

**Affiliations:** 1 Laboratory of Demographic Data, Max Planck Institute for Demographic Research, Konrad-Zuse-Str. 1, DE-18057 Rostock, Germany; 2 Max Planck - University of Helsinki Center for Social Inequalities in Population Health (MaxHel Center), Rostock, Germany; 3 Independent Research Group of Lifespan Inequalities, Max Planck Institute for Demographic Research, Konrad-Zuse-Str. 1, DE-18057 Rostock, Germany; 4 Research Area of Ageing, Mortality and Population Dynamics, Federal Institute for Population Research (BiB), Friedrich-Ebert-Allee 4, DE-65185 Wiesbaden, Germany; 5 Research Group Mortality, Federal Institute for Population Research (BiB), Friedrich-Ebert-Allee 4, DE-65185 Wiesbaden, Germany

**Keywords:** Life expectancy, Causes of death, Germany, High-income countries, Cardiovascular diseases, Divergence

In their letter to the editor, Stolpe and colleagues [1] provide comments on our recently published article “The underwhelming German life expectancy” [2]. The authors raise three issues: (1) impact of quality of cause-of-death statistics; (2) choice of comparison countries and outcomes; and (3) relevance of non-health-care-related factors affecting life expectancy.

The first point concerns an important and well-known issue of the international comparability of cause-of-death data. The authors question the importance of cardiovascular mortality in the persisting German longevity disadvantage against many other Western countries. The claim that research using large groups of causes of death (even whole ICD chapters) produces incomparable results is discouraging and not a position shared by many researchers working on international comparisons of mortality. This proposition challenges the theoretical foundations of epidemiology based on cause-of-death analyses, such as the health transition theory [3]. It also questions the validity of a large number of high-profile studies as well as the usefulness of international databases providing cause-specific mortality indicators such as the WHO Mortality Database. To our knowledge, the existing scattered studies on the quality of cause-of-death reporting provide mixed evidence. More efforts to conduct nationally representative and internationally comparable methodological studies are needed to assess the magnitude of bias due to coding issues. Relying only on cross-sectional comparisons of cause-specific death rates such as presented by Stolpe et al. [1] and small-scale validation studies is clearly not sufficient to draw definite conclusions about the scale and impact of data quality problems.

Stolpe et al. [1] “doubt that reported age-standardized CVD mortality rates are closely associated with a country’s life expectancy”. In fact, we never claimed that CVD mortality levels are predictive of life expectancy levels. The main purpose of our study was to determine the major contributors to the life expectancy gap between Germany and the best-performing countries. There are multiple pathways to underwhelming life expectancy. Many individual longevity-lagging populations show specific problems such as excess lung cancer mortality among Danish females or drug- and violence-related mortality in the US. Is it realistic to assume that CVD is massively misclassified into such causes of death like violent deaths or cancers? Especially the latter are well coded thanks to functioning cancer registers. More generally, longitudinal analysis has shown a clear association between life expectancy increase, and a redistribution of death from CVD to other causes in high-income countries [4].

Our assumption about the importance of cardiovascular diseases for underwhelming German life expectancy is also supported by other evidence and data. First, long-term excess cardiovascular mortality in Germany is consistent with a delayed health transition [4]. Second, we discussed the systematic German disadvantage in other CVD-related metrics – for example, CVD hospitalization and treatment procedure rates that are much higher in Germany. In their commentary on our paper, Baldus and Lauterbach [5] provide additional sobering statistics supporting our argument that CVD-prevention efforts are weak in Germany. It would also be unrealistic to assume that higher rates of CVD-related hospitalization and surgical procedures might be solely attributable to more active treatment strategies in Germany.

The second point raised in the letter concerns the choice of outcome measure (mortality) and comparison countries. In our view, there is a consensus that mortality-based indicators are the most comparable and reliable indicators of population health. Stolpe and colleagues [1] propose healthy life expectancy as an alternative measure. However, health indicators are often based on subjective health measures, which can be partially explained by country-specific reporting behavior and survey design (and its changes). While comparison to additional countries will undoubtedly enrich the nuance, we stand by our original choices which reflect a mixture of longevity vanguards and laggards in the group of high-income countries.

The third point suggests that we ignored the relevance of non-health-care-related factors affecting life expectancy. In fact, our original article discusses non-medical factors, including early life conditions (cohort effects), behavioral risk factors, and regional inequalities [2]. However, we conclude that precise identification of those multidimensional factors is hindered by a lack of nationally representative and internationally comparable data. This conclusion does not imply that these factors do not play a role. We also agree that various social and cultural characteristics (social or family networks, lifestyles, and value orientations) may be directly or indirectly associated with national mortality levels. But grasping the strength of these mechanisms requires longitudinal data.

Stolpe and colleagues [1] conclude that modern life expectancy vastly depends on various determinants which are not associated with health policy. This conclusion is based on a narrow definition of health policy and health systems. Disease prevention is not only an aspect of concern for medical practitioners and policy-makers. Knowledge diffusion from experts to the general public about healthy behavior has played a key role in reducing the disease and mortality burden during the longevity revolution, and health policy has played an important role in fostering this diffusion. Evidence-based and best-practice health policies recognize and address the social determinants of health. Yet besides urgently addressing gaps in prevention and needs for better population-level evidence, more efforts should be directed towards better understanding medical and non-medical determinants of population health in Germany.

We are glad that our work was positively perceived by policy-makers [5], and stimulated further discussion on the topic [1]. While we recognize that cause-of-death classification is not free from errors, it is undeniable that Germany still has high levels of CVD mortality, especially in comparison with vanguard countries. This offers further potential for reduction of the well-established modifiable risk factors associated with CVD deaths. Indeed, reducing CVD mortality requires better data for monitoring, and joint efforts of policy-makers, researchers, and caregivers [5], ideally paralleled by behavioral changes in society. That Germany’s longevity shortfall compared to vanguard countries can mostly be attributed to CVD mortality does not rule out the potential to further reduce the disease burden by tackling other illnesses and risk factors. Given limited resources, it is important to prioritize where progress can be made most effectively.
